# Galanin and galanin receptor expression in neuroblastic tumours: correlation with their differentiation status

**DOI:** 10.1038/sj.bjc.6600019

**Published:** 2002-01-07

**Authors:** Y Perel, L Amrein, E Dobremez, J Rivel, J Y Daniel, M Landry

**Affiliations:** Laboratory of Differentiation and Development Biology, EA DRED 483, University of Bordeaux 2, 146, rue Leo Saignat, 33076 Bordeaux Cedex, France; Onco-Hematology Unit, Department of Paediatrics, Children's Hospital, Place Amelie Raba-Leon, University Hospital, 33076 Bordeaux Cedex, France; Department of Pathology, Place Amelie Raba-Leon, University Hospital, 33076 Bordeaux Cedex, France

**Keywords:** galanin, receptor, neuroblastic tumour, neuroblastoma, differentiation, sympathetic nervous system

## Abstract

Neuroblastoma and its benign differentiated counterpart, ganglioneuroma, are paediatric neuroblastic tumours arising in the sympathetic nervous system. Their broad spectrum of clinical virulence is mainly related to heterogeneous biologic background and tumour differentiation. Neuroblastic tumours synthesize various neuropeptides acting as neuromodulators. Previous studies suggested that galanin plays a role in sympathetic tissue where it could be involved in differentiation and development. We investigated the expression and distribution of galanin and its three known receptors (Gal-R1, Gal-R2, Gal-R3) in 19 samples of neuroblastic tumours tissue by immunohistochemistry, *in situ* hybridization and fluorescent-ligand binding. This study provides clear evidence for galanin and galanin receptor expression in human neuroblastic tumours. The messengers coding for galanin, Gal-R1 and -R3 were highly expressed in neuroblastoma and their amount dramatically decreased in ganglioneuroma. In contrast, Gal-R2 levels remained unchanged. Double labelling studies showed that galanin was mainly co-expressed with its receptors whatever the differentiation stage. In neuroblastic tumours, galanin might promote cell-survival or counteract neuronal differentiation through the different signalling pathways mediated by galanin receptors. Finally, our results suggest that galanin influences neuroblastoma growth and development as an autocrine/paracrine modulator. These findings suggest potential critical implications for galanin in neuroblastic tumours development.

*British Journal of Cancer* (2002) **86**, 117–122. DOI: 10.1038/sj/bjc/6600019 www.bjcancer.com

© 2002 The Cancer Research Campaign

## 

Neuroblastomas (NB) and neuroblastic tumours (NT) are the most common extracranial malignant tumours in childhood (Brodeur *et al*, 1993). These embryonal malignancies derived from the sympathoadrenal lineage of the neural crest show a wide spectrum of differentiation, maturation and organization of neuroblasts and Schwann cells. Thus, at one end of the spectrum there are undifferentiated NT or NB and at the other end there are the fully differentiated, mature NT or ganglioneuromas (GN), passing through transitional states or ganglioneuroblastomas (GNB) (Shimada *et al*, 1999). Most patients with localized NT can be cured with minimal treatment (Brodeur *et al*, 1993), whereas, in contrast, children over the age of 1 year with widely disseminated disease usually have a fatal outcome (Brodeur *et al*, 1993; Coze *et al*, 1997). The spontaneous differentiation and regression of NB reported in some infants is a salient and a unique feature in human oncology (Brodeur *et al*, 1993). This differentiation/regression phenomenon is similar to programmed cell death as described in the normal development of the sympathetic nervous system (Kogner, 1995) and has been demonstrated to be a valuable target for therapeutic use (Matthay *et al*, 1999).

The clinical virulence, differentiation and regression of NT are reflective of the biologic heterogeneity of the tumour. Genetic abnormalities such as MYCN amplification, which are associated with tumour aggressiveness (Bowman *et al*, 1997; Rubie *et al*, 1997), inhibit the regression of NB (Weiss *et al*, 1997). The neurotrophin signals, especially those acting through nerve growth factor (NGF) and its TrkA receptor play an important role in regulating the regression or differentiation of NB (Nakagawara and Brodeur, 1997). NB synthesize and secrete a variety of neuropeptides. The different molecular forms of neuropeptide Y were associated with NB growth (Bjellerup *et al*, 2000), whereas high expression of pancreastatin, vasoactive intestinal peptide and somatostatin were associated with a differentiated neuronal phenotype and a favourable prognosis (Kogner *et al*, 1995; Albers *et al*, 2000). The sst2 somatostatin receptor is considered as a primary target to develop potent neuropeptides analogues for NB therapy (Borgstrom *et al*, 1999).

Galanin is a 29 or 30 amino acid peptide with wide-ranging effects, especially within the neuroendocrine and the central or peripheral nervous system (Bartfai *et al*, 1993). Accounting for the different biological effects, three distinct G-protein-coupled galanin receptor subtypes termed Gal-R1, Gal-R2 and Gal-R3 have been cloned in human (Habert-Ortoli *et al*, 1994; Kolakowski *et al*, 1998). Experiments in animal models suggest a role for this peptide within the sympathetic nervous system during its development (Garcia-Arraras and Torres-Avillan, 1999). No data regarding the distribution and the role of galanin in the human sympathetic system is available so far. In a preliminary study, a highly variable level of galanin expression has been shown in three NB samples (Tuechler *et al*, 1998).

Hence, we further investigated the expression and the distribution of galanin and galanin receptors in NT tissue by using morphological approaches and addressed the role of this neuropeptide in NB differentiation.

## MATERIALS AND METHODS

### Patients

Nineteen patients were investigated. All the 19 children had newly diagnosed and previously untreated NT. The diagnosis of NT was made by histological assessment of a surgically resected tumour specimen. The tumours were classified according to the grading system of Shimada *et al* (1999); (1) NB, Schwannian stroma-poor (UD: undifferentiated, PD: poorly differentiated, D: differentiating); (2) GNB (nodular or intermixed) and (3) GN, Schwannian stroma-dominant. The MYCN copy number was determined by Southern blot analysis. Patients were staged according to the International Neuroblastoma Staging System (INSS) (Brodeur *et al*, 1993). Patients were treated according to protocols described previously (Coze *et al*, 1997; Rubie *et al*, 1997).

### *In situ* hybridization

#### Preparation of probes

Altogether eight oligonucleotide probes (Eurogentec Bel SA, France) were used in this study for *in situ* detection of galanin mRNA (*n*=2), Gal-R1 mRNA (*n*=2), Gal-R2 mRNA (*n*=2), Gal-R3 mRNA (*n*=2). Sequences were complementary to the nucleotides 60–107, 139–186 encoding for human preprogalanin (McKnight *et al*, 1992), to nucleotides 6–53, 984–1031 of the mRNA encoding the human Gal-R1 (Habert-Ortoli *et al*, 1994), to nucleotides 91–138, 1001–1048 of the mRNA encoding the human Gal-R2 (Kolakowski *et al*, 1998) and to nucleotides 41–88, 921–968 of the mRNA encoding the human Gal-R3 (Kolakowski *et al*, 1998).

All oligonucleotides were chosen in regions presenting few homologies with sequences of related mRNAs, and they were checked against the GenBank database.

For radioactive *in situ* hybridization, oligonucleotides were labelled as previously described (Dagerlind *et al*, 1992) at the 3′-end using terminal deoxynucleotidyl transferase (TdT) (Amersham, Amersham, UK), in a cobalt-containing buffer with ^35^S-dATP (New England Nuclear, Boston, MA, USA), to a specific activity of 1–4×10^9^ c.p.m. μg^−1^ and purified by ethanol precipitation. For non-radioactive *in situ* hybridization, 100 pmol of each oligonucleotide probe were labelled at the 3′-end with digoxigenin-11-dUTP (Boehringer Mannheim, Mannheim, Germany), according to published protocols (Schmitz *et al*, 1991).

#### *In situ* hybridization procedure

Human NB samples, frozen immediately after sampling, were cut in a cryostat (Microm, Heidelberg, Germany) and then processed as described earlier (Broberger *et al*, 1997), with 0.5 ng of each of the radioactively labelled galanin or galanin receptor and, when performing double *in situ* hybridization, 2 nM of the digoxigenin-galanin probes.

All sections were then dipped into Ilford K5 nuclear emulsion (Ilford, Mobberly, Cheshire, UK) diluted 1:1 with distilled water, exposed for three (galanin) and eight (galanin receptor) weeks, developed and fixed. Some sections were counterstained with cresyl violet. After mounting in glycerol, the sections were analyzed in a Zeiss Axiophot 2 microscope equipped with a dark field condenser.

#### Quantification

The neuronal profiles were delineated on counterstained sections and the number of silver grains per cell were counted automatically on an image analyser (Histo 200, Biocom, Les Ulis, France) as previously described (Le Moine and Bloch, 1995; Landry *et al*, 2000). To compare the intensity of galanin and galanin receptor mRNA labelling according to differentiation status, grain counting was performed in samples representative of the two ends of the NT spectrum of differentiation, i.e. NB (*n*=7) and GN (*n*=4). Three sections were counted in each case. Owing to their nodular or focal heterogeneity, GNB were not taken in account for grain counting. Results were expressed as a number of grains per μm^2^. Data were presented as mean±standard error; statistical comparisons were made using the Student *t*-test.

### Immunohistochemistry

The NB samples were immersed in 4% paraformaldehyde for 90 mn and rinsed for at least 24 h in 0.1 M phosphate buffer (pH 7.4) containing 10% sucrose, 0.02% bacitracin (Sigma, St. Louis, MO, USA), and 0.01% sodium azide (Merck, Darmstadt, Germany). The samples were sectioned in a cryostat. The sections were then incubated with antiserum at 4°C for 24 h. Polyclonal galanin antibodies (1 : 1000; Peninsula, Belmont, CA, USA) raised in rabbit were detected with biotin-conjugated goat anti-rabbit (1 : 200; Vector Laboratories, Burlingame, CA, USA) and streptavidin-fluorescein (1 : 200; Vector). Monoclonal N-Cam antibodies (1 : 1000; Tebu, Le Perray en Yvelines, France) raised in mouse were detected with rhodamine (TRITC)-conjugated donkey anti-mouse IgG antibody (1 : 200; Jackson Immunoresearch, West Grove, PA, USA). Sections were mounted in an anti-fading solution and examined in a Zeiss Axiophot 2 fluorescence microscope.

### Receptor binding

Binding to galanin receptors was investigated using a fluo-ligand conjugate, fluo-Gal™ (Advanced Bioconcept, NEN™ Life Science Products, Zaventtem, Belgium), consisting of a fluorophore (fluorescein) and the 30 residue human galanin. This peptide was designed to bind with high affinity to human Galanin-R subtypes (Mellentin-Michelotti *et al*, 1999). Cryostat sections were rinsed twice for 5 mn at room temperature in Neurobasal medium (Gibco) containing 0.09% glucose, 0.2% Bovine Serum Albumin (BSA) and 0.02% bacitracin. The sections were preincubated for 10 mn at 4°C, then incubated for 60 mn at 4°C with 20 nM fluo-Gal™ in the same Neurobasal medium solution. The slides were then rinsed and fixed by incubating in 4% paraformaldehyde for 20 mn at room temperature. After mounting in para-phenylenediamine (Sigma), the slides were immediately visualised in a Zeiss Axiophot 2 fluorescence microscope.

### Controls

The specificity of *in situ* hybridization results was established for each mRNA species by incubating four control sections with a hybridization cocktail containing an excess (100-fold) of unlabelled probe.

Specificity of the immunostaining patterns was demonstrated by preabsorption of the antiserum with the corresponding peptide (Peninsula).

The specificity of fluorescent binding was assessed, on serial sections, by the ability to compete the binding of fluo-Gal™ using an excess (100-fold) of unlabelled peptide (Peninsula).

As a control, immunohistochemical studies were also performed on three paediatric tumours : Wilms' tumour (*n*=1), schwannosarcoma (*n*=1), and schwannoma (*n*=1).

## RESULTS

### Characteristics of NT

Age of the patient, location of the tumour, staging and histological classification are summarized in Table 1

**Table 1:**
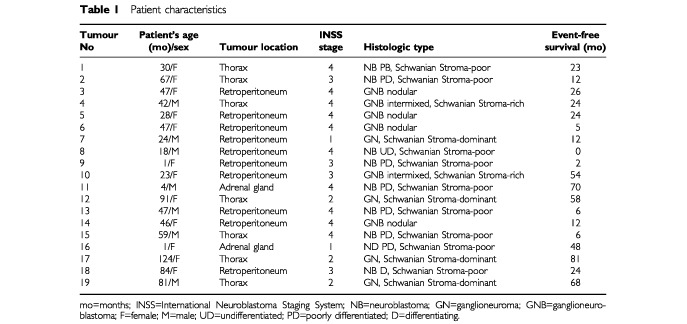
Patient characteristics

. Out of 19 tumours, nine were NB stroma-poor (one undifferentiated, seven poorly differentiated, one differentiating), six were GNB (two of the intermixed subtype, four of the nodular one), and four were GN (Schwannian stroma-dominant) (Table 1). The median age at diagnosis was 46 (range, 1 to 124) months. The median follow-up period after diagnosis was 24 (range: 2-81) months.

### Expression of galanin

**Figure 1:**
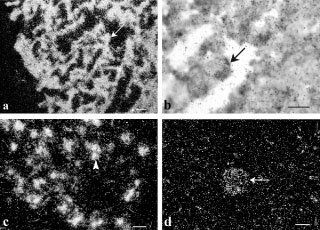
Dark-field (**A**,**C** and **D**) emulsion dipped autoradiograms of NT sections after hybridization with ^35^S-labelled probes complementary to galanin mRNA. The NB contained a high density of strongly labelled small round-cells (arrow) (**A**). In NB, PD, galanin mRNA-containing cells (arrowhead) were organized in rosette-like structures and no significant signal was seen over Schwannian stroma cells (**C**). In GN, a few labelled large neuronal cells (arrow) were detected (**D**). Brightfield micrograph (**B**) of a section processed for double *in situ* hybridization visualizing galanin mRNA using non-radioactive probes and Gal-R1 mRNA, using radioactively- labelled probes; co-localization between galanin and gal-R1 mRNA (arrow). Bars indicate 10 μm.

Galanin messenger was detected by *in situ* hybridization in all tumours investigated (Figure 1A,C and D

). Cresyl violet counterstaining showed silver grains overlying the cytoplasm of neuronal cell bodies (data not shown). No significant signal was seen over Schwannian stroma cells (Figure 1C,D). Galanin mRNA was found in the vast majority of neuroblasts whatever the differentiation stage. A very high number of positive small round-cells was observed in NB (Figure 1A) whereas only a few labelled large neuronal cells were detected in GN (Figure 1D), in accordance with the decrease in cell density. Galanin mRNA-containing cells were organized in dense clusters in NB (Figure 1A) or in rosette-like structures in tumours with early neuronal differentiation (Figure 1C), and they appeared sparse within the stroma of GN (Figure 1D).

**Figure 2:**
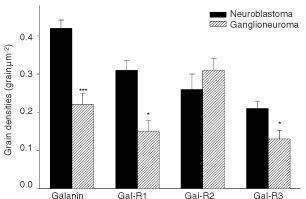
Quantitative evaluation showing the grain densities of labelled neuronal cells after *in situ* hybridization for galanin mRNA and galanin receptor mRNA in NB and GN. Statistical analyses were carried out by using Student *t*-test. *P* value of each counting is indicated. *P<0.05. ***P<0.001.

Grain counting showed that the cellular intensity of the labelling was lower in GN than in NB (0.22±0.03 grains μm^−2^ *vs* 0.42±0.02 grains μm^−2^, respectively, *P*<0.001) (Figure 2

). No significant differences were noticed between metastatic and non-metastatic NT, or according to the age of the patient or the MYCN status (data not shown).

**Figure 3:**
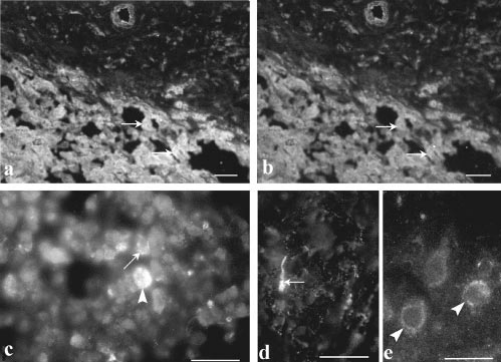
Immunofluorescence micrographs showing Galanin-IR (**A**,**C**–**E**) and N-Cam-IR (**B**). Double immunohistochemistry in NB showed a total overlap of galanin-IR and N-Cam-IR (arrows) (**A**,**B**). Intensely labelled neuroblasts were seen in GNB, nodular, nodular portion (arrowhead) together with weakly positive ones (arrow) (**C**) within the same section. Galanin-positive fibres (arrow) were detected in GNB, intermixed (**D**). The number of galanin-IR containing cells decreased with the neuronal cell density (**A** *vs* **E**). Bars indicate 50 μm.

Galanin immunoreactivity (-IR) was seen in all tumours investigated. Sections processed for double immunohistochemistry showed a total overlap of galanin-IR and N-Cam-IR (Figure 3A,B

). As in the *in situ* hybridization study, the number of galanin-IR containing cells decreased with cell density (Figure 3A vs 3E). In GNB, the intensity of the signal was highly variable from one cell to another with intensely labelled neuroblasts coexisting with weakly positive ones within the same section (Figure 3C). Moreover, some fibres were obviously positive in GNB (Figure 3D).

### Expression of galanin receptors

**Figure 4:**
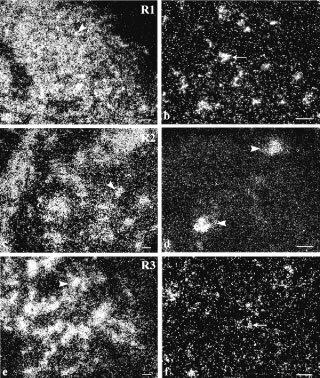
Dark-field micrographs of emulsion-dipped autoradiograms of NT tissue after hybridization with ^35^S-labelled probes complementary to galanin receptor mRNA. The NB contained a high density of strongly labelled small round-cells (arrowheads) (**A**,**C** and **E**). In GN, a weak Gal-R1 and Gal-R3 mRNA labelling was detected in the few large neuronal cells (arrows) (**B**,**F**) whereas a strong Gal-R2 labelling was observed in neuronal cells (arrowheads) (**D**). Bars indicate 10 μm.

Transcripts coding for all three galanin receptors investigated in this study were found in NT (Figure 4

). NB exhibited a homogeneous expression of Gal-R1 (Figure 4A), -R2 (Figure 4C) and -R3 (Figure 4E) mRNAs throughout the whole sample. In GN, galanin receptor messengers were confined to the scattered neuronal cells, not overlying Schwannian stroma cells (Figure 4B,D and F). Furthermore, the cellular intensity of galanin-R1 and -R3 labelling was intense in NB and decreased in GN (Figure 2: R1: 0.31±0.03 grains μm^−2^ *vs* 0.15±0.03 grains μm^−2^ respectively, *P*<0.05; R3: 0.21±0.02 grains μm^−2^ *vs* 0.13±0.02 grains μm^−2^ respectively, *P*<0.05). In contrast, galanin-R2 mRNA levels remained unchanged at the cellular level (Figure 2: 0.26±0.04 grains μm^−2^ *vs* 0.31±0.03 grains μm^2^, *P*: NS). No changes were observed according to the metastatic spread, the MYCN status or the age of the patient.

A high degree of co-localization between galanin and galanin receptor mRNA was found at the various differentiation stages investigated as revealed by double *in situ* hybridization (Figure 1B).

### Galanin binding

**Figure 5:**
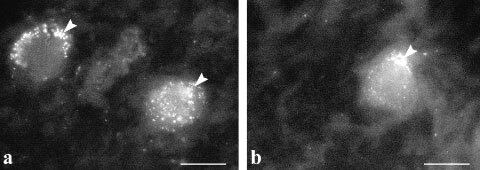
Immunofluorescence micrographs showing fluorescent-galanin binding to receptors in GN. Accumulation of bright spots over the cytoplasm of neuronal cell bodies were seen at the periphery of the perikarya, possibly on the cell membrane (**A**,**B**) or clustered in endosome-like domains located near the membrane (**B**) (arrowheads). Bar indicates 10 μm.

Incubation of sections from GN with fluorescent-galanin resulted in an accumulation of bright spots over the cytoplasm of neuronal cell bodies (Figure 5

). They appeared either scattered at the periphery of the perikarya, (Figure 5A,B) or clustered in endosome-like domains (Figure 5B). In a few cases, fluorescent galanin bound to possible neuronal fibres. In contrast, very weak binding, if any, was detected in NB.

### Controls

No radioactive signal was observed after hybridization with an excess (100-fold) of cold probes. Preabsorption of our antibody with galanin resulted in an absence of labelling. Incubation of fluorescent galanin with an excess (100-fold) of unlabelled peptide totally abolished the signal.

Very weak immunoreactivity, if any, was detected in the three other paediatric tumour samples (Wilms' tumour, schwannosarcoma, and schwannoma). As an internal control, the NT Schwannian stroma cells did not show any galanin-IR, any galanin/Gal-R mRNA labelling or any fluo-Gal™ binding.

## DISCUSSION

This study provides clear evidence for galanin and galanin receptor expression and regulation in human NT.

Galanin was previously reported to be expressed in small cell lung cancer (Sethi *et al*, 1992). Galanin-IR was detected in some adrenal pheochromocytomas but neither in extra-adrenal paragangliomas nor in medullary carcinomas of the thyroid, in endocrine tumours arising in the lung, pancreas, or the gastro-intestinal tract (Sano *et al*, 1991). Galanin was also shown to be released by tumoral corticotropes but not by other pituitary adenomas and appeared to be involved in tumour growth and to be specifically regulated (Invitti *et al*, 1999). Galanin was demonstrated to bind to its specific receptors in human pituitary tumours (Hulting *et al*, 1993). The present findings provided a new example of a neural crest-derived tumour which can be modulated by a strong and regulated expression of galanin/galanin receptors.

In NT, galanin neurone-specific expression was demonstrated by *in situ* hybridization counterstaining and immunohistochemical co-localization with N-Cam, a neuronal marker. Our data do not suggest any relation with the metastatic potential of NT, or with the MYCN status, perhaps owing to the limited number of patients investigated in our study. On the other hand, the present study demonstrates that galanin/galanin receptor expression is related to the differentiation stage, according to Shimada's classification. During embryogenesis, galanin was widely expressed in differentiating neurones of the peripheral nervous system but dramatically decreased postnatally and was no longer observed in mature principal sympathetic neurones of avian models (Barreto-Estrada *et al*, 1997; Garcia-Arraras and Torres-Avillan, 1999), as well as in rodent (Xu *et al*, 1996) and human (Marti *et al*, 1987; Del Fiacco and Quartu, 1994) primary sensory neurones. Similarly, in NB, most undifferentiated neuronal cells displayed robust galanin expression while its levels were reduced in the well-differentiated GN neuronal cells.

Furthermore, neuronal cultures of galanin knock-out mice demonstrated that galanin and its receptors play a critical developmental role and function together with differentiating factors such as NGF in a molecular cascade to regulate regeneration and neuronal cell survival (Holmes *et al*, 2000; O'Meara *et al*, 2000). In the sympathetic ganglion, injury-induced galanin overexpression was triggered by deprivation of NGF (Zigmond and Sun, 1997). Similar mechanisms promoting tumoral cell survival or counteracting neuronal differentiation may be also involved in NB where NGF/TrkA interaction induced differentiation of tumour cells (Eggert *et al*, 2000).

The role of galanin in tumoral cell proliferation or survival may be exerted in an autocrine/paracrine fashion (Invitti *et al*, 1999). This hypothesis is further supported in our study by the co-localization between galanin and its receptors. In animal models, direct paracrine effects have already been demonstrated in differentiating and regenerating neurones (Zigmond and Sun, 1997; Holmes *et al*, 2000), in the pituitary and in various brain nuclei (Todd *et al*, 1998). In several human cancers, autocrine or paracrine overactivation of neuropeptide G-protein-coupled receptors, including those of galanin, could contribute to neoplasia (Heasley, 2001; Marinissen and Gutkind, 2001). Moreover, some neuropeptide analogues induce apoptosis in cancer cells where neuropeptide autocrine and paracrine systems are involved (Heasley, 2001).

GN show specific features, since they display a relatively high level expression of Gal-R2 and a clear signal for fluo-Gal binding. Hence, galanin effects might be exerted through Gal-R2 in differentiated tumoral cells. In contrast, given their high level of expression, all the three galanin receptors could be considered in NB. However, the lack of binding suggests the absence of available receptors at the cell surface. In a previous study, Tuechler *et al* (1998) also suggested that galanin binding was inversely related to galanin peptide concentration in NB tissue. The exact significance of this regulation remains to be elucidated.

The different transduction pathways specifically associated with the different galanin receptors may selectively account for distinct biological activities, including cell-survival and neurite-outgrowth, and for neuropeptide-stimulated growth of cancer cells (Heasley, 2001). The biological activities of Gal-R1 and -R3 receptors are mediated by Gi/G0 G-proteins, leading to the inhibition of adenylate cyclase (Kolakowski *et al*, 1998). In our study, their associated signalling pathways may have played a role in the poorly differentiated NT (or NB). In contrast, Gal-R2 biological activity is exerted through activation of Gq G-protein and phospholipase C and may be involved in processes prominent during nervous system development (Burazin *et al*, 2000). Gal-R2 expression in GN indicates that its associated signalling pathway might also be involved in NT cell differentiation. Thus, galanin receptors may be a useful therapeutic target in neuroblastoma; in human cancers involving neuropeptide receptors system, novel molecules have been defined to selectively induce/inhibit one of the G-protein signalling (Heasley, 2001).

The present data suggest that galanin and its receptors are involved in abnormal differentiation processes of NT. Further studies are warranted to assign specific roles to the different galanin receptors in the modulation of the tumour phenotype and to identify the clinical implications of the expression of galanin in NT.
